# Integrated morphological and transcriptomic analysis of sucker development in *Octopus minor*

**DOI:** 10.1080/19768354.2026.2638580

**Published:** 2026-03-04

**Authors:** Yeonji Kim, Chan-Jun Lee, Kyoung-Bin Ryu, Yam Prasad Aryal, Seonmi Jo, Dae-Cheol Seo, Ji-Hoon Song, Byeong-Gil Jeong, Mi-Jin Lee, Shin-Hong An, Seung-Hyun Jung, Hae-Youn Lee, Sung-Jin Cho

**Affiliations:** a Department of Biological Sciences and Biotechnology, College of Natural Sciences, Chungbuk National University, Chungbuk, Republic of Korea; b National Institute of Wildlife Disease Control and Prevention (NIWDC), Wildlife Disease Response Team, Gwangju, Republic of Korea; c Division of Developmental Biology, Cincinnati Children's Hospital Medical Center, Cincinnati, OH, USA; d Marine Bioindustrial Research Division, National Marine Biodiversity Institute of Korea, Seocheon, Republic of Korea; e Jeollanam-do Ocean & Fisheries Science Institute, Resources Creation Research Institute, Jido-eup, Republic of Korea

**Keywords:** Cephalopods, sucker, Muscle development, comparative transcriptome analysis, WNT signaling

## Abstract

Coleoid cephalopods are excellent models for evolutionary and developmental studies due to their centralized nervous system, short life span, and sophisticated sense organs. Arm suckers, essential for predation, manipulation, and locomotion, have been studied in Decapodiformes, but little is known at the cellular and molecular levels. Here, we investigated sucker development in *Octopus minor* from the embryo to the juvenile stage using morphological, histological, immunostaining, scanning electron microscopy (SEM), in situ hybridization, and transcriptomic analyses. SEM revealed that suckers initially form symmetrically and later become asymmetrical through an embedding process. Histology showed progressive structural differentiation, while immunostaining with acetylated α-tubulin and phalloidin visualized nerve fiber and muscle development. Transcriptome profiling of embryonic stages 12, 14, and 20 identified 2,349 differentially expressed genes (DEGs) linked to arm and muscle development. Among them, *Omi-Gata4*, *Omi-Mef2A*, and WNT signaling molecules (*Omi-Fzd9*, *Omi-Wnt2*, *Omi-Wnt5*) exhibited increased expression in arms and developing suckers, consistent with in situ hybridization results. These findings suggest that WNT signaling contributes to sucker muscle development in *O. minor*, paralleling its conserved role in vertebrates. This study provides new insights into the embryonic morphogenesis of arms and suckers, highlighting the molecular and cellular mechanisms that shape cephalopod appendages.

## Introduction

Cephalopods are classified into two subclasses, Nautilodea and Coleoidea (Decapodiformes and Octopodiformes), based on their external structure and evolutionary trajectory (Bather 1888; Kröger et al. 2011), and belong to the phylum Mollusca, one of the most diverse groups within lophotrochozoan. There are approximately 1,000 cephalopod species, with around 700 marine species inhabiting environments ranging from the deep ocean to tidal waters (Guzik et al. 2005; Kröger et al. 2011; Packard 1972). In particular, the arms and suckers are involved in locomotion, prey capture, and oviposition, thereby contributing to adaptation to their environment (Boletzky 1986; Boletzky 2006; Nixon 1977; Nixon 1985; Ponder and Lindberg 2008). A few studies have investigated the developmental process of cephalopod arms and suckers in Decapodiformes (Kimbara et al. 2020; Nödl et al. 2016; O'Dor et al. 1982; Shigeno et al. 2001; Staaf et al. 2011; Tarazona et al. 2019; Watanabe et al. 1996). However, the development of arms and suckers in Nautilidae, which lack suckers, and in Octopodiformes, remains poorly understood (Sasaki et al. 2010). In addition, the development of several components, such as nerve fibers and vasculature, and their associated signaling pathways, has not been investigated.

Cephalopods possess multiple long arms and tentacles, unique appendages found only in decapods. While the development of appendages has been extensively studied in vertebrates and insects, where it occurs along a proximal-distal axis, involving the interaction of two crucial distal components, the Apical Ectodermal Ridge (AER) and the Zone of Polarizing Activity (ZPA) (Fernández Terán and Ros Lasierra 2008; Niswander 2003; Towers and Tickle 2009; Zeller et al. 2009). The formation of arms in cephalopods also follows a distinctive process. During early development, cell proliferation leads to the formation of a cell mass that outlines the initial arm structure, which then grows along the distal-proximal axis (Nödl et al. 2015). Cephalopod suckers typically consist of three main components (Hou et al. 2012; Miserez et al. 2009). First, the infundibulum (Inf) attach to external substances, such as ground or prey, and possesses a proteinaceous ring that supports the teeth along its rim. Second, the acetabulum (Ac) constitutes the chamber responsible for generating suction; however, in some species, hooks replace this structure, resulting in a loss of suction function (Nixon 1977). Finally, the peduncle connects the arm surface to the Ac. In adults, proximal suckers are larger than distal ones, but their structures are identical (Grasso 2008; Smith 1996). In addition, the formation and structural organization of the sucker primordia proceed in a proximal to distal direction in cuttlefish and squid (Kimbara et al. 2020; Tarazona et al. 2019).

The common long-arm octopus (*Octopus minor*, NCBI taxon ID: 515824) serves as an excellent model for evolutionary and developmental studies due to its highly centralized nervous system, relatively small size, and short life cycle of approximately one year. *Octopus minor* (*O. minor*) have four pairs of arms of varying lengths, each lined with suckers along the oral surface. These arms are capable of performing a wide range of behaviors, including bending, twisting, extending, capturing prey, exploring, and manipulating objects (Hanlon and Messenger 2018; Kennedy et al. 2020). For instance, the first arm is often used to capture small animals hiding in mudflats, while the third arm on the right in males functions as a copulatory device during reproduction (Gutnick et al. 2023). The morphology of octopus appendages has been shaped by evolutionary adaptations to different lifestyles (Boletzky 1999; Boletzky 2003; Shigeno et al. 2008). Additionally, variations in sucker structures and the number of sucker rows have been reported among species in the order Decapodiformes (Kimbara et al. 2020; Nixon 1977). However, our understanding of the dynamics of arm and suckers development during embryogenesis at the cellular and molecular levels in Octopodiformes remains limited.

In this study, we characterized the developmental process of the Octopodiformes from the embryonic stage to the juvenile stage. We examined the development of arms and suckers during embryogenesis using Scanning Electron Microscopy (SEM), histological and immunostaining analysis. Additionally, we identified muscle-forming signaling pathways in the suckers through comparative transcriptomic analysis and *in situ* hybridization. Our findings provide a comprehensive overview of Octopodiformes arm embryonic development and suggest that WNT signaling is involved in this process.

## Materials and methods

### Sample collection and embryo preparation

Embryos of *O. minor* were obtained by rearing cocoons, provided by the Jeollanam-do Ocean & Fisheries Science Institute (Sinan, Jeollanam-do, Korea), in an artificial incubation system maintained under constant darkness (18°C and 33‰ salinity) (Ryu et al. 2023). After each embryonic stage was separated from the yolk, the embryos were fixed in 4% PFA and stored at 4°C. For long term storage, embryos were stored at −20°C after dehydration through a methanol gradient (30∼100%). All experimental procedures were conducted in accordance with animal ethics guidelines, with efforts made to minimize both the number of animals used and their suffering (Boyle 1991; Sakaue et al. 2014).

### Histological sectioning and staining

Cryosection samples (5um) were dehydrated in a sucrose gradient (15% and 30%), embedded in O.C.T. compound (VWR), and rapidly frozen in liquid nitrogen (Jung et al. 2018; Lee et al. 2023). After melting the slide samples for 1 h at 55°C, sections were stained with Mayer’s hematoxylin for 40 s and eosin Y for 3 min. The slides were mounted using Organo Mount (Limonene) after dehydration and clearing.

### Molecular cloning and riboprobe synthesis

Target sequences were amplified with an initial denaturation at 98°C for 5 min, followed by 36 cycles of 98°C for 30 s, sequence-dependent annealing, and extension at 72 °C. Amplified sequences, obtained using gene-specific primers (Supplementary Table1), were subcloned into pGEM-T easy vectors. Linear templates in the plasmid were re-amplified using SP6 and T7 primers, and antisense riboprobes were synthesized using the MEGAscript kit (Ambion, Austin, TX, USA) (Pyo et al. 2025; Ryu et al. 2023).

### Immunofluorescence and phalloidin staining

To visualize F-actin in the *O. minor* arm, whole embryos were incubated overnight with Texas Red-conjugated phalloidin (1:50 in blocking solution; T747, Invitrogen, Thermo Fisher, USA). For immunohistochemistry, sectioned samples were blocked overnight in blocking solution (11096176001, Sigma, St. Louis, MO, USA). Thereafter, samples were incubated with mouse anti–acetylated α-tubulin (1:500 in blocking solution; T-7451, Sigma, St. Louis, MO, USA) or rabbit anti–VEGF receptor 1 (1:500 in blocking solution; ab2350, Abcam, Cambridge, MA, USA) for 24 h at 4°C. After washing with PBT (1× PBS + 0.1% Tween 20), samples were incubated overnight with secondary antibodies diluted in blocking solution (1:500; Alexa Fluor 488 Goat anti-mouse, ab150113, Abcam, Cambridge, UK, or Alexa Fluor 568 Goat anti-rabbit, A11011, Invitrogen, Thermo Fisher, USA). Nuclei were counterstained with DAPI (4′,6-diamidino-2-phenylindole dihydrochloride) solution (1:1000; D1306, Thermo Fisher Scientific, Waltham, MA, USA) and mounted with Fluoromount-G (Southern Biotech, Birmingham, AL, USA).

### RNA isolation and library construction for transcriptome profiling

RNA from *O. minor* embryo arms was extracted using TRIzol reagent (Ambion, Austin, TX, USA) and purified according to the manufacturer’s protocol, then stored at −75 °C. The extracted RNA was submitted to DNAlink (Seoul, South Korea) for library preparation.

RNA libraries were prepared using the TruSeq Stranded Total RNA Kit (Illumina, Inc., San Diego, CA) after rRNA depletion with Ribo-Zero. Library quality and purity were assessed using a NanoDrop 2000C spectrophotometer (Thermo Scientific, Waltham, MA) and an Agilent 2100 Bioanalyzer (Agilent Technologies, Palo Alto, CA). Libraries were quantified using quantitative PCR and TapeStation D1000 ScreenTape (Agilent Technologies, Waldbronn, Germany). Sequencing was performed on the Illumina NovaSeq 6000 platform to generate paired-end 101 bp reads.

### Bioinformatic analysis of RNA-seq data

Read quality was assessed using FastQC (Babraham Bioinformatics, Babraham Institute, Cambridge, UK), and adapter sequences were trimmed with Trimmomatic, version 0.39 (Bolger et al. 2014; Kim et al. 2018). The cleaned reads were then aligned to the *O. minor* reference genome using HISAT2, version 2.2.1 (Cho et al. 2024; Pertea et al. 2016). Gene expression was then quantified in fragments per kilobase of transcript per million mapped reads (FPKM), and differentially expressed genes (DEGs) were identified using the Cufflinks package, version 2.2.1 (Lee et al. 2024; Trapnell et al. 2013; Trapnell et al. 2010).

### *In situ* hybridization and signal detection

*In situ* hybridization was performed following a modified protocol (Aryal et al. 2024; Pyo et al. 2025; Ryu et al. 2023). Briefly, fixed embryos were permeabilized with 0.5 mg/mL proteinase K (Biofact, Daejeon, South Korea), and 4 ng/mL riboprobes were hybridized at 64°C for 48 h. Subsequently, samples were blocked with anti-DIG/POD (1:1000) at 4°C for 16 h, followed by signal detection using a cyanine-3 (1:100) tyramide signal amplification (TSA) kit (PerkinElmer, Wellesley, MA, USA).

### Scanning electron microscopy for morphological observation

The tissues were post-fixed in 1% Osmium tetroxide and dehydrated through an ethanol series and isopentyl acetate. Subsequently, the dehydrated samples were dried in the dark and sputter-coated. Images were photographed using a Field Emission Scanning Electron Microscope (ULTRA PLUS) at the Center for Research Facilities of Chungbuk National University Joint Laboratory.

## Results

### Morphological staging and sucker formation during *O. minor* embryogenesis

**Figure 1.**
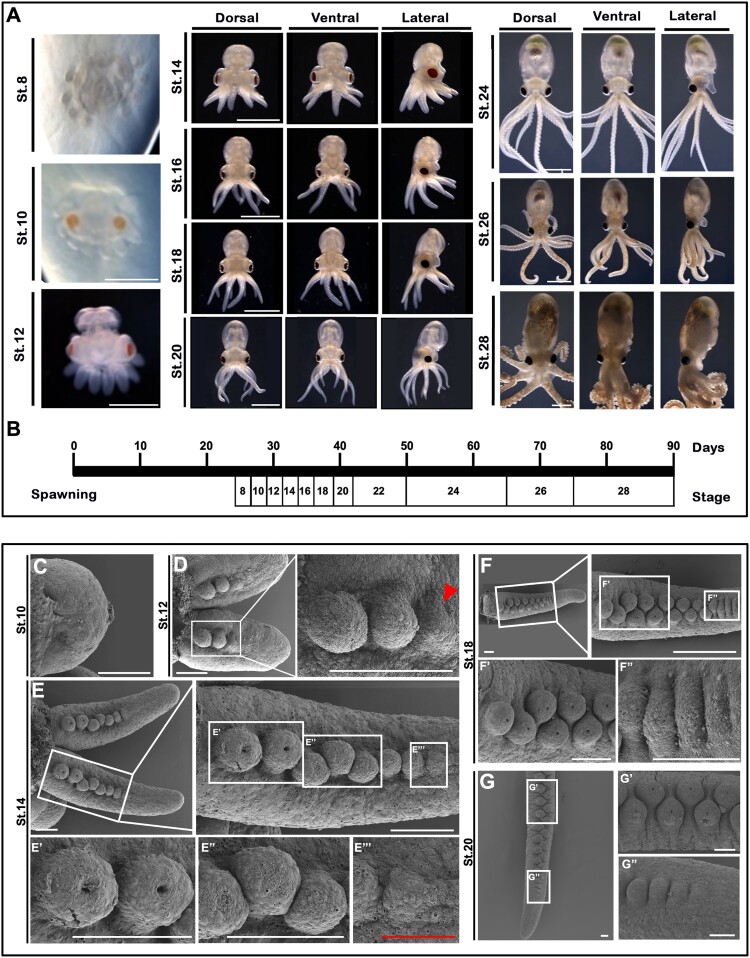
Developmental staging and morphological analysis of *O. minor*. Images showing embryonic stages of *O. minor* at Stage 8, 10, 12, 14, 16, 18, 20, 24, 26 and 28 (A). Timeline of developmental stages of *O. minor* from spawning (stage 1: day 0) to hatchling stage (stage 28: day 90) (B). SEM images of arms observed from the oral side (C-G). The suckers on the inside of the arm have not yet formed at stage 10 (C). Morphogenesis of the arm is first observed as a slight swelling, then progresses into proximo-distal outgrowth at stage 12. Red arrowhead indicates the small swollen area (D). From the proximal region, the sucker begins to develop from a ground-type unit into a mature sucker structure at stage 14 (E). Stages 18 and 20 showing mature sucker structures, and the arrangement of suckers becomes more asymmetric from proximal to distal direction (F-G). High magnification of proximal direction sucker (E’-G’, E”). High magnification of distal direction sucker (E’’-G’’, E’’’). White scale bars: 100 µm. Red scale bars: 50 µm.

The developmental stages of *O. minor* were categorized up to stage 28, from spawning (stage 1) to the juvenile stage (Figure 1(A-B)). Embryo staging was based on morphological characteristics, as described in previous reports (Ryu et al. 2023; Shigeno et al. 2015). Briefly, at developmental stage 8, the embryos are positioned within the yolk, and their arms form clusters around the body. During this stage, the embryos exhibit peristaltic movements of the yolk, and the eye primordia become apparent, though they are not yet pigmented. By stage 10 (28 days after laying), the embryo’s position shifts from the lower part of the yolk to the upper part, and the arms are firmly attached to the body without suckers. The eyes begin to show orange pigmentation. At stage 12 (30 days after laying), mantle formation initiates, though it does not completely cover the internal organs, and the eyes gradually darken from brown to black. At stage 14, the systemic heart and branchial heart are observable, and a heartbeat is confirmed. Arm movement is first detected at stage 16 (35 days after laying). By stages 18–20 (36–42 days after laying), the embryos exhibit pigmented internal organs, active arm movements, and clearly demarcated brain and optic lobes. From stages 22–28, the skin becomes noticeably darker, and camouflage behavior is observed. After stage 24, the embryos are considered juveniles, and by stage 28 (90 days post-fertilization), the juveniles closely resemble adult specimens morphologically, except in size. A developmental timeline was established from stage 8 to 28, covering the period from spawning (day 0) to the juvenile stage (day 90).

SEM was conducted to examine the external morphology of arms during embryogenesis (Figure 1(C∼G)). At stage 10, limb buds are formed, but suckers are not observed on the arms at this stage (Figure 1(C)). By stage 12, the *O. minor* arms have elongated compared to stage 10, and primordial suckers first appear on the proximal side (Figure 1(D)). At this stage, a sucker-producing area is visible at the distal part of the arm as a small swollen region, with band-shaped sucker buds arranged in a row proximally (Figure 1(D); Red arrowhead). At stage 14, the apical invagination of several suckers progresses, and the initially straight arrangement gradually shifts to an asymmetrical structure, beginning to form a zigzag pattern (Figure 1(E)). As development continues to stage 18, the number of sucker buds increases, forming two rows. Band-shaped sucker buds line up in a row at the distal part of the arm (Figure 1(F)), while dome-shaped sucker buds are alternately arranged in two rows at the proximal part (Figure 1(F)). In the stage just before hatching (stage 20), suckers at various developmental stages can be observed along the distal-proximal axis of the arms (Figure 1(G)). Based on these developmental stages and morphological data, we focused on the sucker formation process during stages 10–20, when arm formation initiates, and organogenesis is nearly complete. Among these stages, we excluded stage 18 because it could not be clearly distinguished from stage 20 based on the sucker distribution pattern and morphology. Based on this, we considered the arm patterns in stages 18–20 to be identical.

### Histological characterization of tissue differentiation and sucker morphogenesis in developing *O. minor* arms

**Figure 2.**
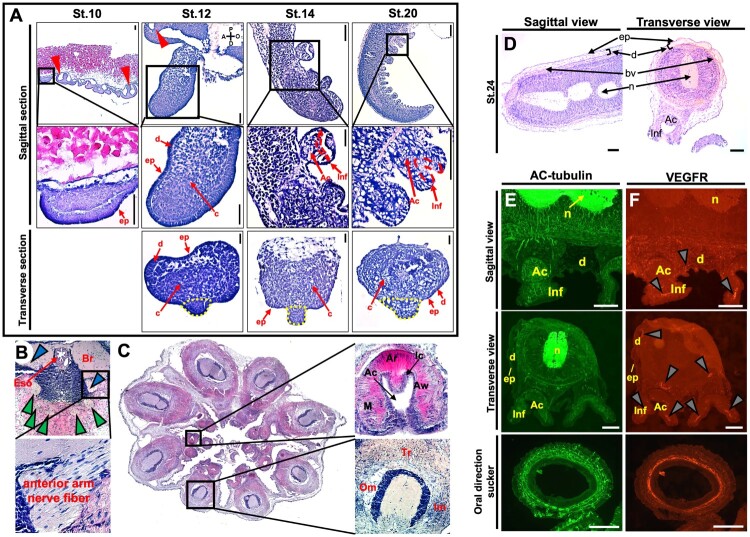
Histology and immunostainings of embryonic and juvenile stages. H&E-stained histological section of the arm part of *O. minor* (A). Red arrowhead: eye primordia. Black box: High magnification area. Red line: Invagination site in the sucker. Yellow line: Sucker region. H&E staining showing cross-sectional view of *O. minor* juvenile (Stage 24). Blue arrowhead: anterior arm nerve fiber. Green arrowhead: posterior arm nerve fiber (B). Histology of a cross-sectional view of eight arms in *O. minor* juvenile (C). The sectional view of arm in *O. minor* juvenile (D). Localization of AC-tubulin (green) in the arm and sucker of *O. minor* (E). Localization of VEGFR (red) in the arm and sucker of *O. minor.* Gray arrowhead: the distribution of VEGFR (F). Black scale bar: 10um. Red scale bar: 50um. Abbreviations: opL, optic lobe; Br, brain; R, retina; Eso, esophagus; om, oblique muscle; tr, trabeculae; lm, longitudinal muscle; ac, acetabulum; inf, infundibulum; ar, acetabular roof; aw, acetabular wall; ic, inner connective tissue layer; m, meridional muscle; ep, epithelium; d, dermis; bv, blood vessel; n, neuropile.

To examine the composition and distribution of internal tissues in the *O. minor* arm, a histological analysis was performed using H&E staining across different stages of *O. minor* embryos (Figure 2(A)). At stage 10, the mantle, eyes, and arms were observed. At this stage, the arm structure is simplified to a single epithelial layer and a cell mass, and the sucker has not yet been identified. By stage 12, an additional layer, the dermis, forms between the epithelial layer and the cell mass, and sucker primordia become visible. At stage 14, the end of the sucker located in the proximal direction of the arm invaginates to form the infundibulum (Inf) and acetabulum (Ac). This structure became more clearly defined at stage 20 and also began to appear in suckers located more distally. At the juvenile stage (stage 24), the histological structures of *O. minor* embryo are more advanced and well-developed. The cross-sectional view at this stage revealed the brain, esophagus, and nerve fibers (Figure 2(B)). It also indicates that the nerve fibres in the arm originate from the brain (Figure 2(B); arrowhead).

Additionally, this stage displayed well-developed eight arms with suckers (Figure 2(C)). The arm structure consists of longitudinal muscles, trabeculae, and an internal oblique muscle layer. The suckers are composed of the acetabulum, acetabular roof, acetabular wall, an inner connective tissue layer, and meridional muscles (Kier and Stella 2007). Both sagittal and cross-sectional views showed that the arm is covered by a simple epithelium and an underlying thick, fibrous dermal connective tissue layer containing blood vessels (Figure 2(D)). To examine the distribution of nerves and blood vessels, we examined the localizations of AC-tubulin and VEGFR. Our results showed that a nerve ring and blood vessels are distributed around the sucker (Figure 2(E and F)). Intense localizations of AC-tubulin and VEGFR were observed along the nerve ring and blood vessels, respectively.

### Spatiotemporal analysis of muscular and neural differentiation in *O. minor* embryos

**Figure 3.**
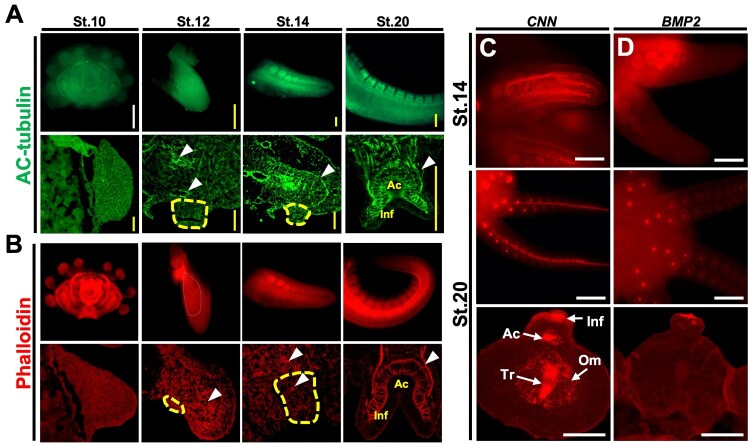
Visualization of nerve fiber and muscle development during embryogenesis. Fluorescence immunohistochemistry was performed to detect acetylated alpha-tubulin (Green) and phalloidin (Red) from stages 10 to 20. Arrowhead: detecting sites. Yellow line: sucker region (A-B). Fluorescent *in situ* hybridization of *O. minor* embryo arms using cyanine3 (Red) (C-D). *Calponin* expression regions were visualized in the whole sample using fluorescent *in situ* hybridization (C). *BMP2* expression regions were visualized in the whole sample using fluorescent *in situ* hybridization (D). White scale bar: 50um. Yellow scale bar: 10um. Abbreviations: Ac, Acetabulum; Inf, Infundibulum; Tr, Trabeculae; Om, Oblique muscle.

Using immunostaining assays with phalloidin and AC-tubulin antibody, we investigated the distribution of muscles and nerves during the embryogenesis of *O. minor* (Figure 3(A-B)). At stage 10, neither antibody was detected. However, after stage 12, both antibodies were detected in the sagittal sections. In stage 12, neither antibody was detected in the sucker, but both were detected in the arm. At stage 14, both antibodies were more clearly detected in the arm and were also observed at the border between the arm and the sucker. In addition, by stage 20, nerve fibers and myocytes extending into the arm are almost fully developed within the sucker. These findings indicate that nerve fibers and myocytes follow largely parallel developmental patterns. Both cell types were initially formed in the arm at stage 12 and gradually extended into the sucker.

F-actin, which can be labeled using phalloidin, is a major component of the cytoskeleton in muscle. In addition, calponin (CNN), which regulates contraction and maintains structure, is widely used as a marker protein for smooth muscle cells. To verify this, we visualized the expression patterns of *BMP2* (PV788799), which are associated with these functions, and *CNN* (PV788801) (Figure 3(C and D)) (Li et al. 2008; Xu et al. 2017). As a result, *CNN* was expressed only in the arms at stage 14. However, by stage 20, it was expressed in both the arms and suckers. This expression pattern was similar to that of phalloidin. In addition, BMP2 associated with calponin function was not expressed at stage 14, but was commonly expressed in the Inf layer at stage 20. Based on these results, we found that by stage 20, smooth muscle–associated molecular features had emerged, and some contractile-related properties had also developed.

### Transcriptomic Profiling Reveals Muscle Formation and WNT Pathway Activation at Stage 20 in *O. minor*

**Figure 4.**
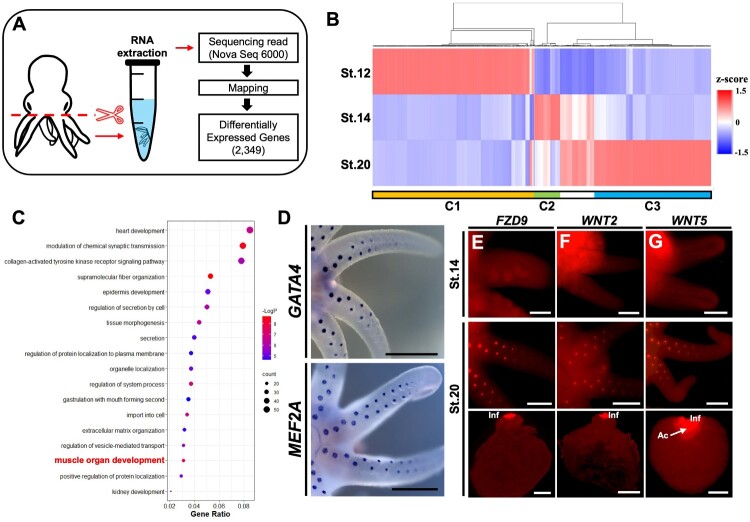
Comparative transcriptome analysis based on muscle-related gene expression patterns. Schematic diagram showing RNA extraction sites and summarizing the DEG analysis pipeline (A). A comparative heatmap of z-scale RNA-seq FPKM values (adjusted *p*-value < 0.05). Red colors denote up-regulation and blue colors denote down-regulation. Hierarchical clustering was done using the Morpheus program (B). Biological Processes GO terms associated with up-regulated DEGs at stage 20 (C). Expression patterns of DEGs associated with the ‘muscle organ development’ GO term at stage 20 (D). Fluorescent *in situ* hybridization of muscle-related Wnt signaling genes in the stage 14 and 20 (E-G). Expression pattern of *FZD9* (E). Expression pattern of *Wnt2* (F). Expression pattern of *Wnt5* (G). Scale bars: 50 µm. Abbreviations: Ac, Acetabulum; Inf, Infundibulum.

Comparative RNA transcriptome analysis was performed to determine whether muscle formation-related Gene Ontology (GO) terms were present at stage 20. To obtain arm and sucker transcriptome data at each stage, the boundary between the head and arm was amputated, and RNA was extracted from the arm (Figure 4(A)). Based on a *p*-value cutoff 0.05, we identified 1,967 DEGs. The heatmap shows a distinct cluster corresponding to each developmental stage (Figure 4(B)). Cluster 1 (C1), which showed high expression at stage 12, included 912 up-regulated DEGs (46.4%). The 149 up-regulated DEGs (7.6%) included Cluster 2 (C2), which were generally highly expressed at stage 14. In the case of Cluster 3 (C3), 680 up-regulated DEGs (34.6%) were highly expressed in the stage 20. We performed GO analysis to determine whether muscle-related Biological Processes were existed in C3 (Figure 4(C)). Result, the ‘muscle organ development’ GO term was identified in C3. Although the gene ratio for this GO term was 0.031 (21 DEGs), it showed statistical significance, with a ‘-log_10_(*p*-value)’ of 7.87. Among this GO term, we identified genes related to muscle development, such as GATA Binding Protein 4 (*GATA4*; PV788803), and maintenance, like Myocyte Enhancer Factor-2 (*MEF2A*; PV788802) (Xin et al. 2006; Zhao et al. 2012). Additionally, both genes were commonly expressed in the suckers on the proximal side of the arm at stage 20 (Figure 4(D)).

Previous studies have shown that MEF2A is involved in both skeletal muscle development and repair, as well as in smooth muscle development (Liu et al. 2014; Shen et al. 2023). In this process, MEF2A modulates skeletal muscle cell growth and differentiation by activating the WNT signaling pathway and directly regulating WNT2 (Liu et al. 2014; Snyder et al. 2013). To determine whether this correlation is conserved in *O. minor* muscle development, we visualized *WNT2* (PV788797) and its representative receptor, *Frizzled-9* (*FZD9*; PV788800), at stages 14 and 20 (Figure 4(E-F)). Although both genes were not expressed at stage 14, they were expressed at stage 20 in the Inf of the sucker on the proximal side of the arm, similar to *MEF2A*. In addition, we visualized another muscle-related WNT gene, called WNT5 (Wang et al. 2024), at stages 14 and 20. Likewise, *Wnt5* (PV788798) was only expressed at stage 20 in the sucker on the proximal side of the arm (Figure 4(G)). These results demonstrate that muscle development and WNT signaling pathway genes are co-expressed at stage 20, when muscle development is evident (Figure 3(B)). Based on these findings, we propose that muscle development pathways, such as WNT signaling, are conserved in the *O. minor* and that muscle formation occurs during the late stages of organogenesis.

## Discussion

This study established the developmental process of *O. minor* from fertilization to the juvenile stage, based on morphological characteristics adopted from previous studies (Ryu et al. 2023; Shigeno et al. 2015), detailing up to 24 developmental stages. While our previous research focused on transcriptome profiling and eye development in *O. minor* (Jung et al. 2018; Ryu et al. 2023), studies on the development of arms and suckers have been lacking. This study revealed the development of arms and suckers in *O. minor* across various embryonic stages, employing a range of experimental methods, including histomorphology, SEM, RNA transcriptome analysis, in situ hybridization, and immunostaining. Embryonic stages from stage 8 to the juvenile stage (stage 24) were analyzed in this study.

Arm development in O. minor begins at stage 10 and fully resembles the adult form by stage 24 (Figures 1 and 2). From stage 8 to 10, O. minor arm buds appeared and exhibited a simple structure consisting of only an epithelial layer and cell mass. However, suckers had not yet formed. The primordia of suckers first appeared at stage 12, when a dermis layer had formed between the epidermal layer and cell mass, and were arranged along the midline of the arm. After this stage, suckers gradually increased toward the distal end of the arm. Additionally, the suckers near the proximal arm region underwent invagination, and their arrangement gradually shifted to an asymmetrical pattern. Based on these results, we found that the development of the arm and suckers was similar to that previously reported in other cephalopods (Anadón 2019; Kimbara et al. 2022; Nödl et al. 2015). During embryonic development, suckers form progressively from the distal end of the arm, and this process depends on directed cell insertion via cell intercalation (Walck-Shannon and Hardin 2014). A similar pattern has been observed during the developmental stages of the kidney and cochlea in vertebrates, and the trachea in flies (Walck-Shannon and Hardin 2014). In addition, we found that nerve fibers and vasculature appear simultaneously in the arm and suckers. Although the two elements were not co-located within the arm during early development, they were closely connected within the Inf layer of the sucker. This neurovascular system is crucial for embryonic development in other organisms, with a similar cell insertion pattern, and we proposed it as an evolutionary and developmental analogue. However, definitive classification of the sucker musculature (e.g. smooth versus obliquely striated) would require ultrastructural analysis.

In this study, we identified that the development of nerve fibers and smooth muscle–associated myocytes occurs simultaneously during embryogenesis (Figure 3(A and B)). Although the two elements developed independently during early embryogenesis, they become co-localized in the sucker by stage 20. It is well known that acetylcholine (ACh), secreted at neuromuscular junctions, induces muscle contraction (Sam and Bordoni 2020). Although we did not detect ACh directly, we identified sequences involved in ACh synthesis and its receptors in the NCBI database. Additionally, *BMP2* and *CNN*, which are involved in the contraction and structural maintenance of muscle cells, are expressed in the sucker at stage 20 (Figure 3(C and D)). Based on these results, we propose that ACh is transmitted through nerve fibers to induce muscle contraction, as is the case in other animals (Rubin 1985; Zhou et al. 2005).

In Figures 1–3, we observed that the suckers became asymmetrically arranged (Figure 1(C-G)) and that the neurovascular system formed between stages 18 and 20 (Figure 2(E-F)). At the same time, we confirmed the formation of muscle fiber and suggested the presence of contraction-related functions. Among these results, we conducted a comparative transcriptomic analysis to identify signaling pathways involved in muscle formation (Figure 4(A and B)). We demonstrated that the GO term ‘muscle organ development’ was detected, and the selected DEGs were expressed in the suckers at stage 20 (Figure 4(C)). Among the selected DEGs, MEF2A is known to upregulate β-catenin expression, thereby activating the WNT/β-catenin signaling pathway. Additionally, dysregulation of the WNT/β-catenin pathway induces developmental defects and disrupts muscle homeostasis (Suzuki et al. 2018; Xiao et al. 2021). Our findings demonstrate that muscle developmental pathways are significantly active at stage 20, and that WNT/β-catenin signaling is involved in muscle development and homeostasis in the suckers (Figure 4(D-G)). Previous studies have shown that WNT and BMP signaling are involved in cuttlefish arm development; however, their role in sucker development remains unclear (Tarazona et al. 2019). Therefore, we demonstrated that the previously unidentified involvement of the WNT signaling pathway is associated with sucker development.

In this study, we provide information on the morphological development of suckers through histological analysis and scanning electron microscopy (SEM). This allowed us to establish a timeline for the appearance of sucker primordia and their subsequent invagination. In addition, we confirmed the development of nerve fibers, vasculature, and myofibers. These findings underscore the intricate relationship between muscle and nerve formation during arm and sucker morphogenesis. Furthermore, comparative transcriptome analysis revealed that WNT/β-catenin signaling plays a critical role in *O. minor* sucker development. These signaling pathways are similar to those involved in myofiber development in both vertebrate and invertebrate muscle, suggesting that the regulatory mechanisms controlling myofiber formation are evolutionarily conserved across Bilateria. In conclusion, our study provides comprehensive insights into O. minor embryonic sucker development and suggests an evolutionary association with muscle-forming mechanisms across Bilateria.

## Supplementary Material

Supplementary Material

## Data Availability

The RNA-seq used in this study have been deposited in the NCBI SRA database under the accession code SAMN49355532 (stage 12 *O. minor* arm RNAseq), SAMN49355533 (stage 14 *O. minor* arm RNAseq), and SAMN49355534 (stage 20 *O. minor* arm RNAseq). Related sequencing information has also been uploaded to NCBI (Supplementary table1).
