# Supplementary material for: Integrated morphological and transcriptomic analysis of sucker development in Octopus minor
Source: Anim Cells Syst (Seoul). 2026 Mar 4;30(1):307–19. doi: 10.1080/19768354.2026.2638580 (PMC12961712; doi:10.1080/19768354.2026.2638580)
Supplement: Supplementary Material [file TACS_A_2638580_SM0539.docx]

| **Gene** | **Accession number** |  | **Primer sequence** |
| --- | --- | --- | --- |
| *Omi_MEF2A* | PV788802 | Forward | 5'-GAATTGGAGCACAAGGCACGT-3' |
|  |  | Reverse | 5'-CTGGCTTGGAATGGCTACGAG-3' |
| *Omi-GATA4* | PV788803 | Forward | 5'-CCTCAACAAGCCCTCCCATT-3' |
|  |  | Reverse | 5'-AGGAGGACTGCCTGATGTCT-3' |
| *Omi-WNT2* | PV788797 | Forward | 5'-GTGCACGAATGATGTGCAGT-3' |
|  |  | Reverse | 5'-TGTATCATAACCTCGCCCGC-3' |
| *Omi-WNT5* | PV788798 | Forward | 5'-TGGAGAGGTATGGCGGAAGA-3' |
|  |  | Reverse | 5'-ATTCTGGCTAATCCACGCGA-3' |
| *Omi-FZD9* | PV788800 | Forward | 5'-TCCGACGTGAAAAACGGCTA-3' |
|  |  | Reverse | 5'-GCCGAGTGGTGGAATATCGT-3' |
| *Omi-BMP2* | PV788799 | Forward | 5'-CAAGGCACAGAAGGGAACGA-3' |
|  |  | Reverse | 5'-ACCAACATACTGAAGCGGCA-3' |
| *Omi-CNN* | PV788801 | Forward | 5'-CGTTTGGGTAGCTCCCTTGT-3' |
|  |  | Reverse | 5'-GGCTTGCAGGCTGGTACTAA-3' |

**Supplementary Table1. *Octopus minor* select gene primer sequence**
